# Application of Gene Knockout and Heterologous Expression Strategy in Fungal Secondary Metabolites Biosynthesis

**DOI:** 10.3390/md20110705

**Published:** 2022-11-10

**Authors:** Yaodong Ning, Yao Xu, Binghua Jiao, Xiaoling Lu

**Affiliations:** Department of Biochemistry and Molecular Biology, College of Basic Medical Sciences, Naval Medical University, Shanghai 200433, China

**Keywords:** fungi, secondary metabolites, gene knockout, heterologous expression

## Abstract

The in-depth study of fungal secondary metabolites (SMs) over the past few years has led to the discovery of a vast number of novel fungal SMs, some of which possess good biological activity. However, because of the limitations of the traditional natural product mining methods, the discovery of new SMs has become increasingly difficult. In recent years, with the rapid development of gene sequencing technology and bioinformatics, new breakthroughs have been made in the study of fungal SMs, and more fungal biosynthetic gene clusters of SMs have been discovered, which shows that the fungi still have a considerable potential to produce SMs. How to study these gene clusters to obtain a large number of unknown SMs has been a research hotspot. With the continuous breakthrough of molecular biology technology, gene manipulation has reached a mature stage. Methods such as gene knockout and heterologous expression techniques have been widely used in the study of fungal SM biosynthesis and have achieved good effects. In this review, the representative studies on the biosynthesis of fungal SMs by gene knockout and heterologous expression under the fungal genome mining in the last three years were summarized. The techniques and methods used in these studies were also briefly discussed. In addition, the prospect of synthetic biology in the future under this research background was proposed.

## 1. Introduction

Microorganisms can produce a wide variety of SMs (e.g., polyketides, terpenoids, saponins, and non-ribosomal peptides), most of which show good biological activity, such as antibacterial, anti-tumor, and immunoregulation properties [1,2,3]. The research on SMs from prokaryotes (such as *Actinomyces*, *Streptomyces*, and other bacteria) started earlier and more thoroughly, while the research on SMs from eukaryotes is relatively scarce. However, with the in-depth study of fungi in recent years, it has been shown that fungi possess more potential to produce SMs than bacteria, and the products have better biological activity, which has attracted more extensive attention and research [4,5]. At the same time, with the ongoing advancement of gene sequencing technology and bioinformatics, a large amount of genomic information on bacteria and fungi has been analyzed and annotated [6,7]. After “mining” this gene information, numerous “silent” biosynthetic gene clusters (BGCs) of SMs from bacteria and fungi still have not been characterized, while fungi show more powerful production potential for SMs than bacteria because of their larger and more complex genomes [8]. The continuous progress of molecular biology technology, such as gene knockout and heterologous expression, as well as the application of combinatorial biosynthesis strategies, makes the manipulation of genes increasingly convenient, which greatly expands the research on microbial SMs [9,10]. All of these promote the biosynthetic pathway study for microbial SMs and show great advantages in the field of biosynthesis.

Gene knockout and heterologous expression are common and mature strategies for the study of the biosynthesis of microbial SMs under the genome mining. The common gene knockout methods mainly include PEG/CaCl_2_-mediated homologous recombination, *Agrobacterium*-mediated transformation, and the CRISPR/Cas technology [11]. In the heterologous expression, the model strains mainly include *Escherichia coli* and *Bacillus subtilis* in the prokaryotes and *Saccharomyces cerevisiae* and filamentous fungi such as *Aspergillus nidulans* and *Aspergillus oryzae* in the eukaryotes [12]. For the prokaryotes, gene knockout and heterologous expression techniques have been well experienced and established because the BGCs of the SMs are usually distributed in clusters and exist in the form of operons, which are convenient for the investigation and operation of target genes (clusters) [13]. For eukaryotes, because of the intact nucleus and genetic system and the larger and more complex genome of fungi, the BGCs of the SMs are usually scattered, which makes it very challenging to dig out and analyze the fungal target genes [14]. Additionally, due to the high level of fungal evolution, it is difficult to establish genetic operating systems, which consequently started late and are relatively scarce in the study of the biosynthesis of fungal SMs [15]. Nowadays, more and more scholars are dedicated to the genome mining of fungal SMs and have obtained a series of achievements. Therefore, it is important and necessary to summarize the research on the molecular biology technology in the secondary metabolites of fungi, especially the gene knockout and the heterologous expression techniques.

In this paper, we primarily concentrated on the representative studies of the biosynthesis of fungal SMs by gene knockout and heterologous expression under the fungal genome mining in the last three years, of which the techniques and methods were briefly introduced. The purpose of this paper is to classify and summarize the strains which have been studied, elucidating the fact that some gene knockout and heterologous expression methods are indeed applicable to the gene manipulation of certain species of fungi, which can provide some ideas and references for future research. In addition, we look forward to the prospect and direction of biosynthesis in the future and to providing new ideas for the biosynthesis.

## 2. Traditional Strategies of Diversity of SMs from Fungi

Microorganisms are important sources of natural products, most of which are isolated from bacteria and fungi. The SMs from fungi have attracted extensive attention and research because of their novel structure (e.g., terpenoids, polyketides, anthraquinones, steroids, and non-ribosomal polypeptides), diverse biological activity (e.g., antibacterial, anti-inflammatory, anti-tumor, and immunoregulation), and rich yield [1,2,3]. 

**Figure 1:**
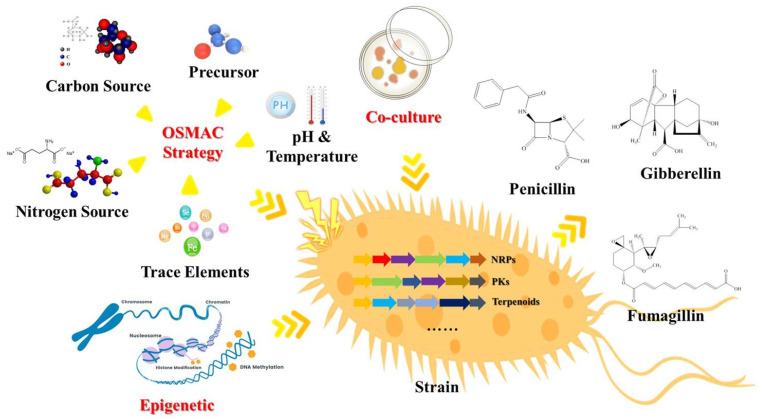
Traditional strategies for fungal secondary metabolites discovery. These mainly include OSMAC strategy, strains co-culture, and epigenetic regulation, aiming to stimulate the expression of biosynthetic gene clusters of SMs through changes of external conditions or exchange of signal molecules between strains in order to obtain more SMs.

Studies on the diversity of SMs from fungi primarily followed the traditional natural product discovery strategy before information on fungal genomes became available (Figure 1). To obtain more SMs with diverse structures and biological activity from the same fungus, the most representative method is the OSMAC strategy. Generally, the same strain may produce various amounts or even distinct kinds of natural products from the different culture conditions (such as the composition of the culture medium, the fermentation conditions, the added precursors, etc.). The scientists have discovered numerous novel and bioactive natural products by the OSMAC method, showing that different culture conditions may activate the silent genes to produce new secondary metabolites [16,17,18]. The OSMAC strategy will continue to be used as a valuable method to exploit the biosynthetic potential of strains. Co-culture, the other traditional natural product mining method, can activate silent genes or clusters through interspecific interactions [19]. In the two-way chemical communications between the co-cultured strains, the signal molecules are transmitted back and forth to interfere with the compound library of co-cultured strains to enrich the quantity of the compounds. Epigenetic regulation is to activate the silent gene clusters through DNA methylation and histone modification without changing the DNA sequence to regulate the secondary metabolic pathway of the strain to obtain new products [18,20]. However, no matter what the traditional method is, the essence is a random selection of the de-silencing of the secondary metabolic pathway, and any conditions affecting the response of a strain to the external conditions may be used to change the transcriptome and then to change the proteome; finally, it can be read out in the variable SMs. This “blind” selection and these changes make it neither possible to accurately understand the law of the biosynthesis of the strains from the perspective of genes, nor to directionally discover the SMs that interested us under the guidance of the genomic information, which still has great limitations in the process of mining natural products.

## 3. Gene Mining and Bioinformatics Broaden the Discovery of SMs of Fungi

With the rapid development of genome sequencing technology, many fungal genome data have been identified and reported, which makes it possible to predict which kinds of compounds may be produced. More and more bioinformatics tools have been developed with the continuous improvement of bioinformatics analysis. BLAST and FASTA are currently the most commonly used database search programs based on local similarity and are tools for the sequence similarity search, which can be used for homologous gene retrieval in public databases [21]. TOUCAN [22] and ARTS [23] can be used for gene mining; antiSMASH [24,25,26] and cluster finder [27,28] were used for analyzing and predicting BGCs; these have greatly promoted the research on fungal SM BGCs. A new stage in the study of the SMs of fungi has been entered. The fungi have large and complex genomes, restricting the analysis of gene clusters. After mining the BGCs of the SMs of fungi, it has been shown that most of the gene clusters (>90%) were unknown [29,30,31]. The genomes of the marine fungi *Calcarisporium* sp. and *Pestalotiopsis* sp. possessed 60 and 67 BGCs, respectively, by bioinformatics analysis, of which the new clusters accounted for 98% and 97%, respectively, and only a small number of BGCs were expressed after RNA-seq verification [30]. Gao et al. reported the high-quality genome sketch sequence of the endophytic fungus *Neonectria* sp. DH2, of which 14,163 genes are predicted to encode proteins, and 557 of the genes are unique. According to the neighborhood-linked phylogenetic tree of the ITS region, there were 47 BGCs in the DH2 genome, of which only 5 BGCs were previously reported, showing the huge production potential of the SMs of fungi [31].

Unlike the traditional research strategy, the study of the fungal SMs based on the gene mining is to associate the SMs with the BGCs by bioinformatics analysis, predict the potential biosynthesis pathway, and then verify the prediction by molecular biology techniques and to analyze the biosynthesis pathway of the SMs. In this research strategy, it is more definite to discover what we want, master the law of biosynthesis, and to develop it more accurately. Gene knockout and heterologous expression have been widely used in the study of fungal secondary metabolic biosynthesis and have played an important role.

## 4. Application of Gene Knockout Strategy in Biosynthesis of SMs of Fungi

**Figure 2:**
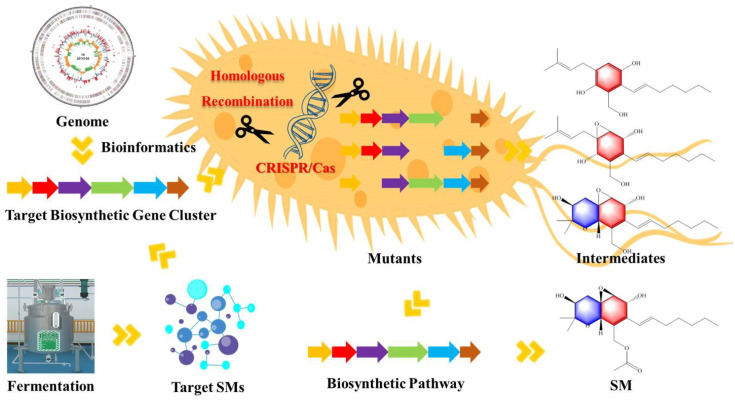
Application of gene knockout strategy in the study of fungal secondary metabolites. It mainly includes the determination of target BGCs, construction of mutants, and identification of intermediate products, which verifies the biosynthetic pathway.

Gene knockout is one of the important methods for studying the biosynthesis pathway of fungal SMs; it mainly focuses on the in vivo verification of the gene function of the studied strains (Figure 2). The studied genes (clusters) are usually predicted to be expressed in the original strain, rather than silent genes. The research idea is usually to determine the existence of interesting compounds in the fermentation broth of the original strain first, then to sequence the gene of the strains, predict the biosynthesis pathway (gene clusters) of the interesting compounds after homologous gene mining and bioinformatics analysis of the genomes, and then verify the correlation between the gene cluster and the biosynthesis of the natural products by the gene knockout. The function of the studied genes in the natural product biosynthesis can be roughly judged by the construction of single or multiple gene mutants and the accumulation of intermediate metabolites, which preliminarily analyzed the biosynthetic pathway of the products.

### 4.1. Application of Different Gene Knockout Methods

Different strains have formed different physiological and biochemical characteristics in the long process of evolution. Even if the evolutionary tree shows that the strains are in the same genus, their morphological, physiological, and biochemical characteristics are also quite different. Therefore, the establishment of the genetic transformation system needs to be investigated and selected according to the actual situation of the different strains. Currently, PEG-mediated homologous recombination, *Agrobacterium*-mediated transformation, and PEG-mediated CRISPR/Cas technology are the main methods used in the research on the gene knockout related to the biosynthesis of fungal secondary metabolites.

#### 4.1.1. PEG-Mediated Homologous Recombination

PEG-mediated homologous recombination is a classic method for fungal gene research, based on the preparation of high-quality protoplasts, which induces foreign DNA into cells by PEG/CaCl2 and other methods. PEG is a cell fusion agent which can interfere with the recognition between cells by causing the disorder of the surface charge of the cell membrane, thus facilitating the intercellular fusion and the entry of foreign DNA molecules into the protoplast. The homologous recombination reaction strictly depends on the homology between the DNA molecules. The homologous recombination reaction is usually based on the formation and resolution of cross molecules or the Holliday junction structure, that is, the precursor stage, the formation of the synaptonemial complex, and the resolution of the Holliday structure.

It has been widely used in the biosynthesis of SMs derived from *Aspergillus*. In knocking out the key genes *GedF* and *GedK* of the anthraquinones biosynthesis from *A. fumigatus*, a revised questin ring-opening mechanism was elucidated; this caused a classic Baeyer–Villiger oxidation hypothesis, which has been challenged [32]. By comparing the metabolites of wildtype *A. fumigatus* and *crmA*, a deleted strain grown under Cu^2+^, it was found that at the level of trace Cu^2+^ CrmA participated in two different biosynthetic pathways to improve the adaptability under environmental pressure [33]. After knocking out the oxepinamides biosynthesis gene derived from *A. ustus*, the necessary intermediates were obtained, and the biosynthesis pathway was analyzed for the first time [34]. Analyzing the azaphilones biosynthesis gene derived from *A. terreus*, synthesized by two independent gene clusters, provides a new idea for the biological mechanism of complex compounds synthesized by filamentous fungi [35]. At the same time, it has also been applied in the study of the biosynthesis pathways of natural products, such as the oxygenated phenethyl derivative from *A. ustus* [36] and the hopane-type triterpenoid glycoside from *A. fumigatus* [37], indicating that it is generally applicable to *Aspergillus* fungi. In addition, for xylomyrocins from *Paramyrothecium* sp., their biosynthesis pathway was identified through gene knockout and stable isotope feeding, which clarified the fusion coordination between carbohydrate metabolism and NRPS skeleton synthesis and enriched the biosynthesis sources of the special assembly units of non-ribosomal peptides [38]. Liu et al. confirmed the biosynthetic gene cluster of sordarin in the *Sordaria araneosa*, proving that four P450 oxidases play an important role in the rearrangement process [39]. The PEG-mediated homologous recombination method has also been used in the study of biosynthetic genes in epidithiodiketopiperazines derived from *Trichoderma hypoxylon* [40] and indolizidine alkaloids derived from *Curvularia* sp. [41], showing that this method has been widely used in gene knockout.

#### 4.1.2. PEG-Mediated CRISPR/Cas Technique

The CRISPR/Cas technique uses RNA to guide the Cas protein to modify the targeted sequences; this has been widely used in various fields as a hot spot. The CRISPR-Cas9 gene editing technology is to identify the target genome sequence through the artificially designed sgRNA (guide RNA) and to guide the Cas9 protease to effectively cut the double strands of DNA to form double strand breaks. The damage repair will cause gene knockout or knock-in and finally achieve the goal of modifying the genome DNA.

In the fungi gene study, CRISPR/Cas technology often requires PEG-mediated protoplast transformation. Scientists knocked out the meroterpenoids biosynthesis gene from marine fungus *Talaromyces purpureogenus* and evaluated two NHI proteins from a heterodimer for catalysis, analyzing the biosynthesis of heteroterpene [42]. Several biosynthetic genes of aculenes derived from *A. aculeatus* were inactivated, which provided reference for the synthesis and derivation of daucane sesquiterpenes [43]. In addition, CRISPR/Cas technology has played an important role in the functional research on phomoxanthone A, derived from marine fungus *Diaporthe* sp. [44], and flavoprotein monooxygenase, derived from *A. terreus* [45]. This technique is becoming mature, with more and more applications in the future study of fungi genes.

#### 4.1.3. *Agrobacterium*-Mediated Transformation 

There are relatively few studies on fungal gene knockout mediated by *Agrobacterium* because of the difficulty of the genetic operation and the low transformation efficiency of the fungi. Mycotoxin patulin isolated from *Penicillium expansum* can cause fruit and product pollution. Li et al. used *Agrobacterium*-mediated transformation to study the patulin biosynthesis gene cluster and confirmed the function of all the genes involved in its biosynthesis, providing the support for the prevention and treatment of pathogenic microorganisms [46]. Zhang et al. knocked out a pyrone meroterpenoid oxalicine B gene cluster from *P. oxalicum* and further elucidated its biosynthesis pathway and oxidase catalytic mechanism through in vitro biochemical verification. Oxalicine B possessed good anti-influenza virus activity [47]. Research on a “super” gene cluster of *Metarhizium robertsii* shows that this cluster contains three secondary metabolic gene clusters. It is confirmed that different gene deletions do not affect the insecticidal virulence of *M. robertsii* but do significantly affect the ability of *M. robertsii* to resist different bacteria because of the different gene deletions leading to the production of different structural compounds [48]. 

### 4.2. Other Applications of Gene Knockout Strategy

Gene knockout could also be applied to the diverse study of SMs in fungi, except for the verification of the function of genes, such as for the activating of silent gene clusters and increasing product diversity. Wei et al. knocked out the key genes in the biosynthesis of rubratoxins, the main product of *P. dangeardi*, which makes it easier to inhibit the production of the main compounds and competitively obtain common precursors of polyketide synthesis and isolate novel skeleton compounds [49]. Qi et al. realized the abundant accumulation of emodin, the precursor of physcion, by knocking out the key emodin-1-OH-O-methyltransferase gene in *A. terreus* [50]. This method could also apply in the agricultural pathogenic bacteria, such as the biosynthesis of toxin ustilaginoidins derived from *Ustilaginoidea virens* [51], the infection of fusaoctaxin B derived from *Fusarium graminearum* on the plant virulence factor [52], and the verification of the multiple gene function in the biosynthesis pathway of penifulvin, an anti-insect compound derived from *P. griseofulvum*, which provides an important reference for the prevention and control of agricultural pathogenic microorganisms and the development of new green biological pesticides [53].

### 4.3. Limitations of Gene Knockout Strategy

There are also many defects and limitations in the study of fungal SM biosynthesis by gene knockout technology. First of all, because of the high level of evolution and incomplete genetic system of fungi, it is difficult for most strains to establish a genetic transformation system by conventional methods. Regardless of the PEG-mediated homologous recombination or CRISPR/Cas technology, they both require the high-quality protoplasts, while the low transformation efficiency is common in the protoplasts [54]. In addition, there were usually a lot of verification works needed in the screening of mutant strains. Secondly, it is difficult to analyze and identify all the metabolites in the fermentation by the current separation and identification techniques because of the unknown metabolic pathways of most of the studied strains and the complex metabolite compositions. Based on this, the gene deletion is easy to ignore in the regulation of biological metabolites. In the process of studying the biosynthesis pathway of mycotoxin flavipucine derived from *A. nidulans*, gene knockout could only determine the protein involved in the synthesis of the toxin. When the single gene in the BGC was knocked out, there were no intermediates observed; this needs to use the heterologous expression strategy to clarify its specific biosynthesis pathway [55]. Gene knockout strategies play the role of “verifier” in most studies focusing on whether genes (clusters) are involved in the synthesis of certain SMs, but they cannot explain how genes participate in biosynthesis exactly. This rough verification can be used as a guiding tool to study the primary stage of fungal SMs.

## 5. Application of Heterologous Expression Strategy in Biosynthesis of SMs of Fungi 

**Figure 3:**
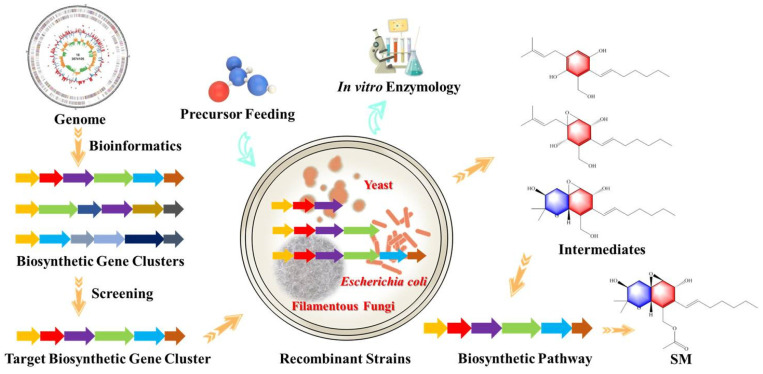
Application of heterologous expression strategy in the study of fungal secondary metabolites. It mainly includes the screening of target BGCs, heterologous expression of recombinant strain, feeding of precursor, in vitro enzymatic verification, and identification of intermediate products, in order to predict the biosynthetic pathway.

Because of the disadvantages and limitations of the gene knockout strategy, the heterologous expression strategy has become the other important method in the study of the biosynthesis of the SMs of fungi. Gene data mining reveals that many fungi possess cryptic BGCs that appear to be silent when cultivated in the conventional fermentation conditions [8]. It is difficult to study the function of these genes by gene knockout when carrying out a detailed study of the biosynthetic pathway of complex SMs, while it is necessary to use heterologous hosts with a clear genetic background and mature genetic transformation system to express these silent or complex biosynthetic genes (clusters) (Figure 3). The general research idea of heterologous expression mainly includes the whole genome sequencing of the original strain, blasting and searching for the homologous genomic data to obtain the gene clusters encoding the biosynthesis of the SMs of interest. After the bioinformatics analysis of these genes (clusters), the meaningful genes (clusters) are cloned. Finally, the genes were heterologously expressed by genetic transformation, and the corresponding biosynthetic pathway was clarified by the identification and analysis of the products. There could be the heterologous expression not only of a single gene, but also of the whole gene cluster. When a single gene was heterologously expressed, it mainly combined with the in vitro enzyme experiments to characterize the expressed proteins in detail; when the gene cluster was heterologously expressed, it mainly combined with precursor feeding to verify the hypothetical biosynthesis pathway.

### 5.1. Application of Different Heterologous Hosts 

The selection of the heterologous host is the key to the successful application of heterologous expression. Firstly, it is important to determine the clear genetic background of the heterologous expression strain. Secondly, the heterologous expression strain should have the advantages of simple culture and fast growth. Finally, the simple and easy manipulation and high transformation efficiency of the genetic transformation were chosen. At present, the mature and commonly used expression systems in the biosynthesis of SMs of fungi include *Escherichia coli* and *Bacillus subtilis* in prokaryotes and *Saccharomyces cerevisiae* and filamentous fungi in eukaryotes, such as *Aspergillus nidulans* and *Aspergillus oryzae*. The selection of model strains should consider the sources of different genes (clusters) and the physical and chemical properties of the expressed products.

#### 5.1.1. Application of Filamentous Fungi as Heterologous Hosts

With the in-depth study of filamentous fungi, there are more outstanding advantages than the model strains with, for example, prokaryotes and yeast as the heterologous hosts. The filamentous fungi can correctly splice introns from heterogenous fungi and express multiple biosynthetic genes in fungi concurrently, which plays a role in protein translation and post-modification to obtain the target product. At present, the filamentous fungi expression systems commonly used in fungal heterologous expression were mainly *A. nidulans* and *A. oryzae*.

As a filamentous fungus, *A. nidulans* has a relatively clear genetic background after years of research, and a relatively mature genetic transformation system has been established. It has been widely used as a model strain in the study of the heterologous expression of the SMs of fungi. With the combined heterologous expression of the biosynthesis gene cluster of sordarinane, derived from *Sordaria araneosa* with enzyme experiments in vitro, a group of P450 multi-enzyme systems was confirmed. The new catalytic function of the P450 family oxidases was demonstrated, which laid a foundation for the development of P450 multi-enzyme synergistic catalysis for the synthesis of new chemical entities [56]. Wei et al. identified the functions of some P450 enzymes and sesquiterpene synthetases by the heterologous expression of the BGC of asperaculin A, isolated from *A. aculeatus* [57]. Due to the failure of the gene knockout strategy, Zhong et al. heterogeneously expressed the BGC of rumbrin, isolated from *Auxarthron umbrinum*, and found an autoantibody gene, verifying that the product had anti-HIV activity [58]. In addition, the BGCs of flavunoidine-1 derived from *A. flavus* were recombinantly arranged and heterologously expressed in *A. nidulans* in different combinations, and the BGCs containing both TC and NRPS core enzymes were identified [59]. In addition, the BGCs of harzianic acid [60] and trichoxide [61] from *Trichoderma*, citridone [62] and ilicicolin H [63] from *Penicillium*, and decarestricitine from *Beauveria bassiana* were heterologously expressed in *A. nidulans*, combined with precursor feeding, which clarified the biosynthesis pathway to some extent, respectively [64].

*A. oryzae*, a heterologous host, has similar characteristics and advantages to *A. nidulans*, which is also widely used as a model strain of biosynthetic genes in the SMs of fungi. In the reconstruction of the biosynthetic pathway of phlegmacins derived from *Talaromyces* sp., an unprecedented laccase-involved unsymmetrically regioselective oxidative coupling reaction was shown, which provides a new reference for the synergistic catalytic mechanism of laccases and other proteins [65]. The BGC of funiculolides derived from *A. funiculosus* were heterogeneously expressed to elucidate the fact that α-ketoglutarate-dependent dioxygenase FncG catalyzed spirocyclopentanone [66]. By the heterologous expression of CJ-20557 biosynthetic gene clusters from *A. duricaulis*, in combination with in vitro enzyme experiments, it was clarified that the SAT domain of polyketide synthase DrcA was responsible for the formation of the depside bond, which enriched the understanding of the mechanism of fungal NR-PKS biosynthesis [67]. Chen et al. confirmed that the enzyme IlIS, a sesquiterpene synthase from *Irpex lactous*, was responsible for the synthesis of the tremulane skeleton through heterologous expression and in vitro enzymatic reaction, and four new tremulane sesquiterpene products were isolated [68]. The specific PKS synthase GrgF in the BGC of gregatin A derived from *Penicillium* sp. [69] and the trichobrasilenol terpene cyclase derived from *Trichoderma atroviride* [70] were verified by the heterologous expression of *A. oryzae*. In addition, the NRPS-PKS gene cluster from *A. candidus* was heterologously expressed to obtain a pyrrolobenzazepine alkaloid [71]. Additionally, conidiogenone [72], brevianamide A [73], and brevione E [74] derived from *Penicillium* were heterologously expressed in *A. oryzae*, combined with the precursor feeding, which is helpful in analyzing the biosynthesis pathway.

#### 5.1.2. Application of *Saccharomyces cerevisiae* as Heterologous Host

Because of the early study of the genetic background and the mature genetic manipulation system, *Saccharomyces cerevisiae* was widely used as the heterologous expression model strain in the study of the SMs of fungi in the past. Eukaryotic *S. cerevisiae* could translate proteins correctly and post-modify. Zhang et al. heterologously expressed *P. funiculosum* source chrodrimanin-type meroterpenoids BGCs and verified them in in vitro catalytic experiments to clarify the function of CdnC protein, obtaining a series of new chrodrimanins compounds [75]. In the biosynthetic path of cyclohexanoid terpenoids derived from *Aspergillus* sp.*,* two key enzymatic functions were characterized by heterologous expression and in vitro enzymology experiments [76]. In addition, *S. cerevisiae* has also been applied for the expression of entire BGCs. *S. cerevisiae* was used as an expression vector in the heterologous expression of the BGC of the formation central C ring in the tetracyclic ergoline derived from *A. fumigatus* [77]. In the study of the biosynthesis pathway of shimalactones derived from *Emericella variecolor* [78], and flavunoidine 1 [59] and diorcinol [79] derived from *Aspergillus, S. cerevisiae* was also the heterologous expression vector. However, compared with filamentous fungi as expression vectors, *S. cerevisiae* still has some shortcomings, such as the lack of an advanced mRNA splicing system and the difficult in expressing complex BGCs; it is only suitable for expression of single or simple BGCs, which restricts its further application in the study of fungal SMs.

#### 5.1.3. Application of *Escherichia coli* as Heterologous Host 

As the earliest heterologous host, *Escherichia coli* has been widely used in genetic engineering, metabolic engineering, and other fields. Because of its advantages, such as simple cultivable, rapid growth, high transformation efficiency, and so on, *E. coli* has a good effect on the heterologous expression of BGCs from prokaryotes. However, it has similar shortcomings with *S. cerevisiae*, lacking the introns splicing of eukaryotes and the post-translational modification process, (glycosylation, phosphorylation et al.), which restrict the heterologous expression of the fungal biosynthetic gene cluster. It is generally used to heterologously express a single gene of BGC and is combined with in vitro enzyme experiments to characterize the protein function. The key genes in the BGCs of nanangelenin A [80], diorcinol [79], and asperaculin A [57], derived from *Aspergillus*, fumiquinazoline [81], and brevianamide A [73], derived from *Penicillium*, and trichobrasilenol, derived from *T. atroviride* [70], were heterogeneously expressed in *E. coli.*, combined with in vitro catalysis and other experiments, which clarified the functions of a series of key enzymes in the biosynthesis pathway.

### 5.2. Application of Heterologous Expression Strategy in Mining Silent BGCs

The rapid development of gene mining and bioinformatics has brought new breakthroughs to the discovery of fungal SMs. A large number of terpenoid, PKS, and NRPS gene clusters have been predicted from the genome sequences of many fungi, most of which are unknown. The actual kinds of obtained secondary metabolites of the strains were far less than the predicted kinds based on the BGCs’ functions because of the gene silence under conventional experimental conditions. How to activate these silent genes to obtain lots of SMs is the current research focus. At present, there are generally two ways to activate the silent gene clusters. One is to overexpress the silent gene clusters by adding strong promoters to or regulating the transcription factors of the original strains. Seven new compounds were obtained by overexpressing the specific transcription factor tenS of *Beauveria bassiana*-derived silent gene clusters [82]. However, it has not been widely used in fungi because of the difficulty in establishing genetic system in the original strain. The other is to clone these silent gene clusters and transform them into model strains for heterologous expression, which is a common research method at present. Nine new sesquiterpenes were discovered after the heterologous expression of silent gene clusters derived from *A. ustus* in *A. oryzae*, which strongly supports the application of heterologous expression in the gene mining of silent BGCs [83].

### 5.3. Limitations of Heterologous Expression Strategy

Although heterologous expression technology has significant advantages in the study of fungal secondary metabolite biosynthesis, it still has some limitations, especially in terms of the compatibility of heterologous strains with foreign genes. The proteins could be expressed and modified well by heterologous host filamentous fungi, but filamentous fungi still have some defects. When the silent hancockinone A BGC, derived from *A. hancockii*, was heterologously expressed in *A. nidulans*, the target product was not obtained due to intron cleavage and inactive expressed protein [84]. Moreover, the genetic stability of the recombinant strain is very important. How to maintain stable genetic characteristics is a problem to be solved. In short, the heterologous expression system still needs continuous technical optimization in order to better serve the biosynthesis of SMs of fungi. 

## 6. Conclusions

With the advent of the post-genome era, the research on SMs has gradually deepened from the characterization of phenomena to the process of mining essence, which gene sequencing and bioinformatics have strongly promoted. The research on the SMs of fungi has entered a new stage. In the study of fungal SMs biosynthesis, gene mining provides a theoretical foundation; molecular biology sets up the essential circumstances, and strategies such as gene knockout and heterologous expression were the methodologies. The further exploration of synthetic biology will exert a profound impact on the biosynthetics of SMs and will continue to promote the discovery process of SMs.

Synthetic biology is the extension and future development direction of biosynthesis research, which is the rebuilding process of the life. It starts from the basic elements to build the parts step by step, hoping to design and synthesize new life processes or organisms based on the human will. It mainly included the construction of chassis cells; the selection and optimization of gene elements; design synthetic pathways, such as gene integration and modular synthesis strategies; and the establishment of cell factories to regulate metabolism, all of which are accompanied by advances in molecular biology techniques and the development of gene editing tools. It is reported that different BGCs of decalin-containing diterpenoid pyrones have been retrieved from five fungal genera, and five natural pathways, one shunt pathway, and four extension pathways were recombined by the selection and integration of genes in *A. oryzae*, producing 15 new compounds, which exhibited the role of synthetic biological methods in enriching the diversity of fungal SMs [85]. Synthetic biology has unique advantages in stabilizing the source and yield of SMs, which is bound to bring new hope for the directional and controllable biosynthesis of SMs.

## Data Availability

No new data were created or analyzed in this study. Data sharing is not applicable to this article.
